# Tip cells: Master regulators of tubulogenesis?

**DOI:** 10.1016/j.semcdb.2014.04.009

**Published:** 2014-07

**Authors:** Helen Weavers, Helen Skaer

**Affiliations:** Department of Zoology, Downing Street, Cambridge CB2 3EJ, UK

**Keywords:** Tip cell, Branching morphogenesis, Tubulogenesis, Cell fate, Intercellular signalling, Collective cell migration

## Abstract

The normal development of an organ depends on the coordinated regulation of multiple cell activities. Focusing on tubulogenesis, we review the role of specialised cells or groups of cells that are selected from within tissue primordia and differentiate at the outgrowing tips or leading edge of developing tubules. Tip or leading cells develop distinctive patterns of gene expression that enable them to act both as sensors and transmitters of intercellular signalling. This enables them to explore the environment, respond to both tissue intrinsic signals and extrinsic cues from surrounding tissues and to regulate the behaviour of their neighbours, including the setting of cell fate, patterning cell division, inducing polarity and promoting cell movement and cell rearrangements by neighbour exchange. Tip cells are also able to transmit mechanical tension to promote tissue remodelling and, by interacting with the extracellular matrix, they can dictate migratory pathways and organ shape. Where separate tubular structures fuse to form networks, as in the airways of insects or the vascular system of vertebrates, specialised fusion tip cells act to interconnect disparate elements of the developing network. Finally, we consider their importance in the maturation of mature physiological function and in the development of disease.

## Highlights

• Single tip cells or groups of leading cells develop at the forefront of growing tissues.
• Tip cells regulate tubule growth and morphogenesis.
• Tip cells develop distinctive patterns of gene expression and specialised characteristics.
• Tip cells are required for health and may be involved in the progression of cancer.

## 1 Introduction

The construction of an organ involves the regulation of many different cell activities including cell specification, proliferation, growth, recruitment, movement, shape change and finally differentiation. Failure to regulate any one of these in time and space has disastrous effects and all need to occur in coordination with the others to produce the final patterned and fully functional structure. While many aspects of developmental control result from reciprocal signalling involving all or the majority of constituent cells, a special class of distinctively placed cells at the tips of tubes or the leading front of migrating cell groups have been found in a wide variety of systems to regulate the activity of their neighbours at key stages of organ development. In this review we discuss the selection and distinctive characteristics of these so-called tip cells and chart their activities and, where known, the mechanisms by which they exert their influence.

**Fig. 1:**
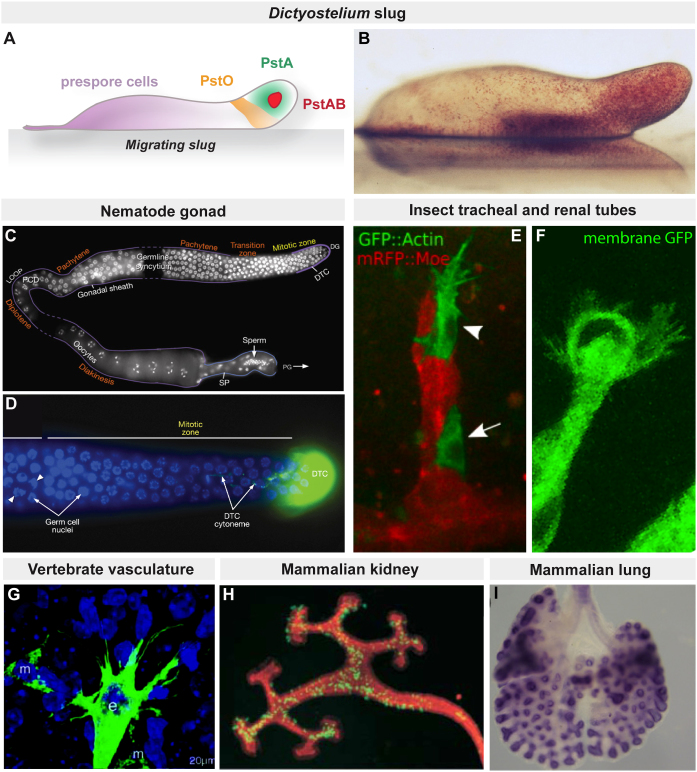
Examples of tip cells. Specialised cells located at the tips of developing organs are found in diverse tissues from primitive *Dictyostelium* slugs (A) to the mammalian kidney and lung (H and I). In the migrating slug (A,B), prestalk A (pstA) cells populate the apical tip and guide slug migration; the remainder of the slug is composed of prestalk cells, pstO cells and pstAB cells. In the *C. elegans* gonad, a single distal tip cell (DTC, green in D) is located at each end of the U-shaped gonad arms at the tip of the mitotic region (C,D). In the insect tracheal (E) and renal systems (F), dynamic tip cells (E, arrowhead) with prominent filopodia are found at the distal-most ends of the developing tubes. Tip cells are also observed in the vertebrate vasculature during sprouting angiogenesis (G). Groups of cells located at the growing bud tips regulate branching morphogenesis in the mammalian kidney (H) and lung (I). Figure credits: images reproduced with permission from (B), D Dormann University College London; (C) and (D), J Maciejowski & E Hubbard NYU from http://www.wormatlas.org; (E), M Affolter University of Basel originally published in Curr Biol doi: http://dx.doi.org/10.1016/j.cub.2008.10.062; (G), C Betsholtz, Karolinska Institute ©Betsholtz et al., 2003. Originally published in JCB doi:10.1083/jcb.200302047; (H), F Costantini Columbia originally published in Dev Cell doi; http://dx.doi.org/10.1016/j.devcel.2004.11.008; (I) V. Papaioannou Columbia from PLOS Genetics 2012 doi:10.1371/journal.pgen.1002866.

Tip cells can only be loosely defined because they occur as a distinctive and more or less permanent cell type at the tip of a wide variety of developing tissues, often a tube or branched structure within an organ. However, they are also found as a collection of cells marked out by their position at the leading edge of a moving group of cells, where they exert an influence over their neighbours. A common feature of tip cells is that they have specialised patterns of gene expression and exhibit specific cell behaviours. They feature from the simplest multicellular organisms (in the migrating slug of *Dictyostelium*) to the most complex (the vascular and renal systems of mammals) and in tubular systems (insect and mammalian airways or renal tubules) as well as in groups of migrating cells (insect border cells, zebra fish lateral line) (see Fig. 1 for examples).

Whilst there are many striking parallels in the molecular mechanisms governing the selection, behaviour and function of cells at the tips of what initially appear to be physiologically and morphologically diverse tissues, there are also crucial differences, which ensure that an organ's structure is tailored for its specific physiological function.

Our aim in this review is to highlight major roles played by tip cells during tubulogenesis and in the mature tissue, taking examples from diverse systems. We do not aim to provide a comprehensive description of tip cell activity in every organ.

## 2 Tip cell specification and selection

In many tissues tip cells are selected by a regulatory network, in which high levels of a facilitating signal confer on a group of cells the potential to develop tip cell fate. This potential is then restricted by competitive and mutual inhibition through Delta-Notch signalling to refine patterning, determining which cell or cells actually adopt the tip cell fate. However the levels of initial signal bias the outcome of lateral inhibition as more highly activated cells inhibit their neighbours more effectively. For example, during angiogenesis in mammalian systems high levels of activating Vascular endothelial growth factor receptor (VEGFR) signalling (VEGFR2/3) and low levels of inhibitory VEGFR1 signalling lead to enhanced expression of the Notch ligand, Dll4, enabling these cells to outcompete their neighbours for the tip cell fate (reviewed in [1,2]). This network appears to be conserved in zebrafish [3–5].

**Fig. 2:**
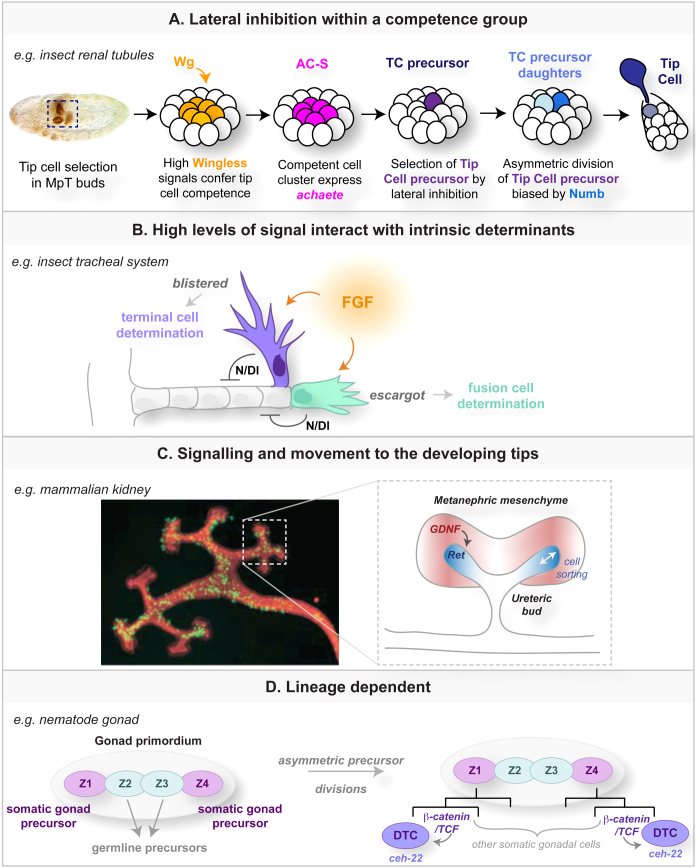
Strategies for tip cell selection. In the insect renal (A) and tracheal (B) systems, high levels of signalling (through the Wnt ligand Wingless in (A) and FGF in (B)) confer tip cell competence and tip cell potential is further refined by lateral inhibition through Notch/Delta. In the renal tubules, this occurs firstly within the competent cell cluster and later between the daughters of the tip cell precursor, where the outcome is biased by asymmetric inheritance of the Notch inhibitor, Numb. In the insect tracheal system, two types of tip cell are specified (terminal cell and fusion cell) according to different gene expression patterns in the progenitors (*blistered* in presumptive terminal and *escargot* in presumptive fusion cells). Conversely in mammalian kidney, cells with highest levels of Ret signalling (activated by GDNF secreted by neighbouring metanephric mesenchyme) sort out and populate the ureteric bud tips (C). Selection of DTCs in the nematode gonad is lineage dependent (D) but the asymmetric divisions of the Z1a and Z4p progenitors are biased by β-catenin/TCF signalling to specify a single DTC in each gonad arm. Image in (C) reproduced with permission from F Costantini Columbia originally published in Dev Cell doi; http://dx.doi.org/10.1016/j.devcel.2004.11.008.

In a very similar way tip cells in the developing tubes of both the tracheal and renal system in *Drosophila* are selected by signals promoting tip cell fate (high levels of Fibroblast Growth Factor (FGF) signalling in trachea and of Wingless and JAK/STAT in Malpighian tubules ([6,7]; Denholm, Brown et al., unpublished)), followed by refinement through lateral inhibition again mediated by Notch and Delta (see Fig. 2A for renal tubules) [8,9]. Analysis of *Breathless* (FGF receptor) clones in developing dorsal tracheal branches indicates that cells receiving higher levels of FGF signalling than their neighbours always acquire tip cell fate but that the final outcome is determined by Notch-mediated competitive interactions. However, Araujo and Casanova [10] shows that, in contrast to the dorsal branches, the Notch/Delta pathway does not act during tip cell selection in the ganglionic branches, indicating that differing levels of FGF signalling might provide sufficient information to discriminate tip *vs.* trailing cell fate. Once specified, tip cells exhibit altered patterns of gene expression, changes in cell shape and in the activity of the cytoskeleton (see [11,12]).

In both the tracheal and renal tubules of *Drosophila*, two types of tip cell are specified (Fig. 2A and B). In the tracheal system terminal tip cells regulate outgrowth and carry oxygen to target tissues in lumen-containing cellular extensions and fusion tip cells join the branches from adjacent segments to form an interconnected network. The distinction between terminal and fusion tip cells is determined by differing patterns of gene expression. Escargot is induced by activation of the Dpp pathway and its targets promote fusion cell fate while repressing terminal cell fate [13]. Terminal cells express the FGF antagonist Sprouty, and the transcriptional regulators Pointed and Blistered [9]. In Malpighian tubules the cell initially selected (tip cell progenitor) divides once (Fig. 2A). Through a second round of Notch-Delta signalling, one daughter takes the tip cell fate, which is marked by the expression of the *achaete-scute* genes and their targets. This cell undergoes a pronounced shape change so that it projects from the distal end of the tubule. The other daughter remains fully integrated in the epithelium but develops a distinctive pattern of gene expression to become the sibling cell [8]. Signalling between the daughters is biased by the asymmetric segregation of Numb, which acts to down-regulate Notch, so that the cell containing it adopts the tip cell fate. Loss of Numb results in the segregation of two sibling cells at the expense of a tip cell and the over-expression of Numb results in the development of two tip cells [7].

Groups of tip cells lead the collective migration of cell assemblies and are found at the forefront of branching systems in vertebrate organs such as the lung and kidney. These tip cells are not always selected at the tip; alterations in their behaviour after selection bring them to the leading edge (Fig. 2C). This was first shown in a non-tubular system, the primitive organism *Dictyostelium*, where cells acquire their fates through both cell intrinsic (cell cycle phase) and extrinsic factors (growth history, receipt of signals such as differentiation inducing factor, DIF, and cAMP; reviewed in [14]). Cells in G2 that enter the slug late in its development receive the highest levels of DIF and become pstA cells. These cells spiral to the apex, populating the leading tip of the migrating slug [15]. In a similar way, cells in the ureteric bud of the vertebrate kidney that receive high levels of Glial cell-line derived neurotrophic factor (GDNF)/Ret signalling undergo extensive movements in a process of competitive cell sorting to generate the ureteric bud tips [16] (Fig. 2C). In postnatal murine mammary glands mosaic inactivation of FGFr2 reveals that cells lacking FGF signalling are competitively excluded from the highly proliferative terminal end buds but are able to contribute to branch trunks [17]. In the mammalian lung localised dynamic expression of FGF10 at branch tips promotes both the proliferation and outgrowth of the bud [18]. Cells at the tip, that receive high levels of FGF10, laterally inhibit their neighbours, through secretion of Bone Morphogenetic Protein 4 (BMP4), preventing them from contributing to the bud [19].

In contrast to these mechanisms for tip cell selection, all of which involve competition between cells that receive different levels of signalling, the specification of the distal tip cells (DTCs) in the nematode *Caenorhabditis elegans* hermaphrodite gonad appears to be lineage dependent (Fig. 2D). The gonad develops two arms, each containing one DTC derived from the somatic precursors Z1 and Z4. The DTCs occupy symmetric positions (Z1.aa and Z4.pp) in each lineage [20,21]. However interactions between cells also play a pivotal role in DTC fate. The final cell division that gives rise to each DTC is asymmetric and is controlled by Wnt/β-catenin signalling [22–24]. The activated daughter becomes the DTC and starts to express *ceh-22* (a Nkx2.5 homeodomain transcription factor). Ectopic expression of either β-catenin or Ceh-22 is sufficient for both daughters to develop as DTCs [25].

Thus in each case tip cell fate results from intrinsic and/or inductive factors that confer tip cell potential and, in most cases, refinement of that potential through competitive interactions.

## 3 Tip cells regulate tubulogenesis

Tip cells regulate tubulogenesis through both tissue-intrinsic and tissue-extrinsic mechanisms, which must be tightly coordinated to generate a physiologically competent organ of appropriate size and shape. In some cases, tip cells play important mitogenic roles, regulating organ growth in a tissue-intrinsic manner, as seen in the nematode gonad and insect renal system (see Section 3.1). Tip cells also play pivotal roles in establishing three-dimensional tube architecture and this is often achieved by interactions with surrounding tissues. Here, tip cells direct branch outgrowth and guide tube migration by responding to extracellular instructive guidance cues and by directly attaching to nearby cells (see Sections 3.2–3.5). In this way, tip cells precisely coordinate tubulogenesis with respect to the development of surrounding tissues. Furthermore, tip cells often remain active post-embryonically (see Section 4), when they regulate changes in organ structure in response to alterations in metabolic activity or the environment, and also function more directly in organ physiology [26–29].

### 3.1 Mitogenic signalling: tip cells pattern cell proliferation

In both the nematode gonad and insect renal system, tip cells play essential mitogenic roles to pattern cell proliferation. Whilst tip cells in the insect renal system function early in embryonic development to generate the mature tubule cell number, the tip cells of the nematode gonad function in post-embryonic development and later in the adult, forming a niche to maintain the germ-line stem cell population.

The hermaphrodite gonad of *C. elegans* has two symmetric U-shaped arms each with a single specialised somatic leader cell, the DTC, at its end (Fig. 1C and D, for review see [30]). The germline is highly polarised along the proximal-distal axis with mitotic stem cells located nearest the DTCs and meiotic cells more proximally. Surgical DTC ablation results in arrest of mitosis and initiation of meiosis in all germline stem cells and premature maturation into gametes, which is normally restricted to cells in the proximal-most regions of the gonadal arms [31–33]. DTCs therefore act to promote mitosis and maintain the stem cell pool distally, whilst restricting germ cell maturation and meiotic entry to more proximal regions. This regulation requires signalling through the Notch pathway; the LAG-2 ligand is expressed in DTCs and the receptor GLP-1 in germline cells, where activation promotes mitosis and blocks meiotic entry [34]. Accordingly it is thought that germ cells become meiotic only when they move out of the range of mitogenic signalling of the DTC.

**Fig. 3:**
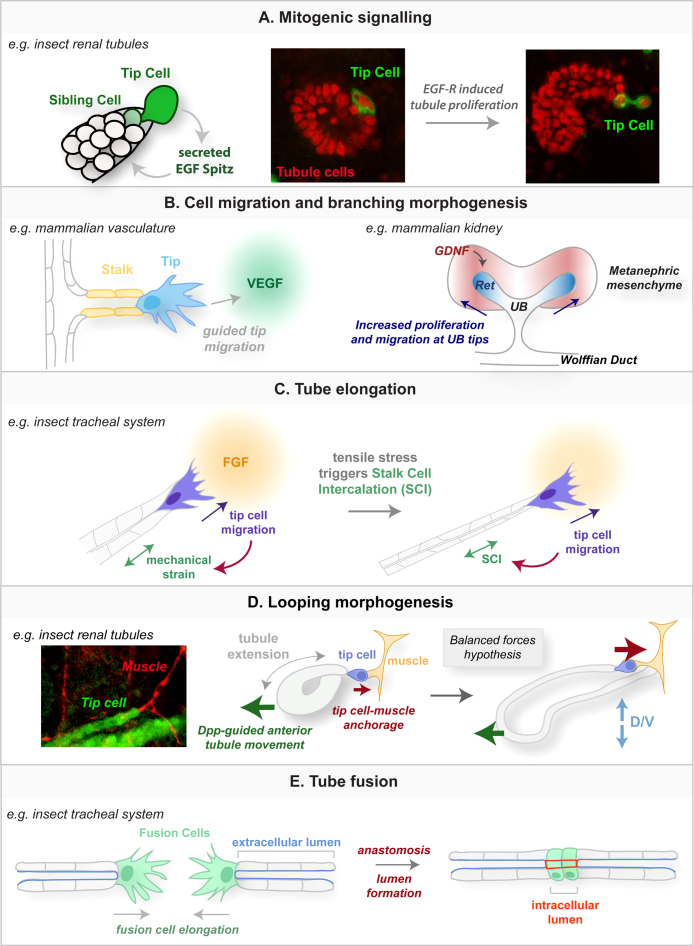
Tip cells regulate multiple steps of organogenesis. Renal tip cells (with their sibling cells) stimulate tube growth by secretion of the EGF ligand Spitz to activate mitosis in neighbouring tubule cells (A). Tip cells also migrate towards chemoattractants, guiding tube outgrowth and branching morphogenesis (B). In the vasculature, tip cells guide newly sprouting vessels towards pro-angiogenic factors such as VEGF, whilst high Ret signalling activated at the tips of the kidney ureteric buds stimulates bud outgrowth. Guided tip cell migration also serves to elongate the growing tubes (C). In the insect tracheal system, tip migration towards FGF sources creates mechanical strain along the tube that induces stalk cell intercalation (SCI) and tube extension. Conversely, tip cells in insect renal systems do not lead tube outgrowth but instead anchor the tube ends to nearby muscles (D). Such anchorage antagonises forward-directed tube movement, ensuring that tubes develop a characteristic looped structure and are positioned stereotypically in the body cavity. Specialised tip cells in the vertebrate vasculature and insect tracheal systems promote tube fusion to create large interconnected tubular networks (E). In the trachea, fusion cells elongate as they migrate towards each other, before finally making contact and establishing a continuous lumen to interconnect the tubes.

Insect renal system tip cells (Fig. 1F) also pattern cell proliferation along the proximal-distal axis of developing Malpighian tubules (Fig. 3A). During embryogenesis, two distinct phases of cell proliferation occur in the tubules to establish the mature number of cells. Initially all tubule primordial cells divide synchronously but later division is restricted to the distal regions and this phase is dependent on the tip cell and its sibling, located at the distal-most end of each growing tubule [35]. Tip and sibling cells secrete the mature Epidermal Growth Factor (EGF) ligand, sSpitz, and activate EGF signalling in neighbouring cells to promote mitotis [36,37]. Removal of the tip cell lineage, either genetically or mechanically, or abrogation of EGF signalling, causes premature arrest of mitosis resulting in short, stunted tubules with dramatically reduced cell numbers [35–38].

The presence of tip cells and their role in regulated cell division of Malpighian tubule cells is not unique to *Drosophila* but is common to the development of a number of insects, in genera separated widely by evolution. For example in the blood-sucking Hemipteran, *Rhodnius*, tip cells are apparent as soon as the tubule primordia appear, and as in *Drosophila*, are required for cell division of their neighbours and remain prominent throughout tubule division and elongation [39].

### 3.2 Tip cell-directed tube migration and navigation

Cells at the leading edge of the growing tubes play important steering functions to guide the developing organ appropriately through the body cavity. In the *C. elegans* gonad, the DTCs not only regulate germline mitotic/meiotic transition, but the DTC-specific migratory route leads arm outgrowth and generates the distinctive U-shaped hermaphrodite morphology ([32,40,41]; see [30] for review). DTCs respond to a complex network of attractive (TGF-β) and repulsive (Netrins) signals from dorsal and ventral body wall muscle, respectively, to guide their migration [42,43]. ECM is remodelled by the metalloproteinases (MMPs), GON-1 and MIG-17, that act at the DTC surface, to clear a pathway for gonad outgrowth [93,44,45]. A conserved signalling network through CED-2 (murine CrkII), CED-5 (*Drosophila* Myoblast City) and CED-10 (Rac1 GTPase) acts in the DTC to control cell shape changes during cell migration [40,46].

Tip cells also play important guidance functions in mammalian systems such as sprouting angiogenesis. Here, pioneering tip cells are found at the outgrowing front of newly sprouting endothelial branches (Fig. 3B). These highly polarised cells exhibit striking filopodial activity in response to angiogenic stimuli such as VEGF; in this way single tip cells within each branch guide the growing vessel towards an angiogenic stimulus [47]. Vessel growth itself is achieved by proliferation of endothelial stalk cells positioned behind the leading tip cells, although tip cells themselves do not divide [48]. Live imaging has revealed a dynamic and regular exchange of tip and stalk cell fates, suggesting that the VEGF/Dll4/Notch signalling network is continuously updated as sprouting vessels encounter new environments [49].

There are instances during organogenesis when groups of cells cohere and migrate as a group, in a process known as collective cell migration [50]. In the *Drosophila* salivary gland, cells at the leading front of the primordium guide gland migration along several tissues to establish their final position within the body cavity ([51] and see Girdler and Roeper, in this issue). These cells direct the posterior migration of the newly invaginated glands along the circular visceral muscle (cVM) of the midgut [52,53]. Leading cells extend dynamic lamellipodial protrusions in the direction of migration along the muscle surface. Integrins and their extracellular matrix (ECM) ligand Laminin appear to be essential for this migration, as mutations in α-PS1 or α-PS2 integrins or α-1,2 Laminin cause defects in tip cell lamellipodia formation and failure in gland migration [52,54]. Later, the salivary glands lose contact with the cVM of the gut but the leading cells act to ensure that the distal end of the gland adheres stably to the longitudinal visceral muscle (lVM) of the gastric caeca, once gland migration is complete [53,55].

### 3.3 Tip cells promote branching morphogenesis

Branching morphogenesis underlies the tubular scaffold of many organs such as the vertebrate kidney, lung, vasculature and insect tracheal system, which arise from the remodelling of epithelial or endothelial sheaths into vast tubular networks. In each of these systems, local signalling between the budding tubule tips and adjacent tissues plays crucial instructive roles in tubule branching and ultimately determines the final branching pattern of the organ.

The branching patterns of the insect trachea and vertebrate vasculature are established in a remarkably similar manner by local guidance cues that induce cell competition and cell migration locally at the tubule tips. In the insect, the FGF ligand Branchless (Bnl) is expressed locally in patches of epidermal and mesodermal cells around the tracheal sacs [56], which is sensed by tracheal cells expressing the FGF receptor Breathless (Btl) [56,57]. Tracheal cells with the highest levels of Btl activity outcompete neighbouring cells for the tip cell fate (see Section 2; [6]), develop dynamic filopodia at their surface and migrate towards the Bnl source. Such tip cell migration distally generates tension within the proximal stalk cells, inducing cell intercalation that elongates the newly forming tubular branches [58]. Similarly in the vasculature, local sources of pro-angiogenic factors, including VEGF ligands, induce cell competition, leading to the emergence of highly active tip cells that throw out multiple filopodia to induce cell migration in vessel tips and new vessel outgrowth (Fig. 3B).

Although distinctive ‘tip cells’ have not been identified in developing mammalian lung and kidney, groups of cells at the tips of outgrowing branches show specific patterns of gene expression and distinctive behaviours. Branching morphogenesis is induced at the tips of primary lung buds and ureteric buds by local reciprocal interactions with surrounding mesenchyme. In the lung, FGF10 is expressed dynamically in mesenchyme surrounding the primary epithelial buds [18,59,60] and activates signalling in the bud tips *via* the FGFr2b receptor, which is required for budding. Cells in the lung bud tips upregulate target genes including *sprouty2, bmp2 & 4 and sonic hedgehog* that act to further refine FGF signalling and FGF10 expression [61–65]. The exact cell behaviours that act downstream of local FGF signalling to induce tube outgrowth remain unclear, although recent studies suggest that oriented cell divisions in the lung epithelium in response to RTK receptor signalling play an important role in branch architecture [66].

The developing ureteric bud (UB) of the vertebrate kidney arises from the Wolffian duct, and undergoes a complex pattern of growth, branching and remodelling to form the urinary collecting duct system [67]. Similar to the lung, the tips of the developing UBs are the major sites of cell proliferation, UB growth and branching [68–70] (Fig. 3B). GDNF is secreted from mesenchymal cells adjacent to the UB [71] and activates its receptor Ret locally at the UB tips. GDNF signalling induces cell proliferation at the bud tips and this precise activation of localised cell division is thought to contribute to bud evagination [70,72,73]. Given that GDNF has been implicated as an important chemoattractant for cultured kidney cells [74], it is possible that GDNF also guides the migration of the UB tips [75].

### 3.4 Tip cell-driven tube elongation by cell intercalation

A major step during tubulogenesis is the dramatic elongation of the primordial buds into the long tubes of the mature tissue. In the insect tracheal and renal systems, the process of tube extension involves cell rearrangements in the plane of the epithelium, in the absence of cell proliferation. Strikingly these cell intercalation events are triggered by cells at the tips of the developing tubes.

As described above (Section 3.2) tip cells of the *Drosophila* tracheal system extend dynamic filopodia and lamellipodia in response to FGF signalling, activated by local sources of the ligand Bnl. This tip cell activity leads to the directed migration of the tracheal branch towards the FGF source (Fig. 3C). Such distal migration generates mechanical tension in more proximal tracheal stalk cells and this promotes stalk cell intercalation (SCI) that further elongates the tracheal tube [58,76,77].

In *Drosophila* renal tubules, postmitotic cells undergo dramatic morphogenetic rearrangements, intercalating between their neighbours to produce a four-fold increase in tubule length and a reduction in cells surrounding the tubule lumen from 8 to 12 to just 2 [78]. Tubule extension occurs in a tissue-intrinsic manner, as embryonic tubules that have been dissected out and cultured *in vitro* elongate normally [35,79]. Recent work shows that tip and sibling cells secrete the EGF ligand Spitz to produce graded activation of EGF signalling along the distal-proximal tubule axis. This orients cells in the plane of the epithelium and promotes polarised cytoskeletal activity, which produces circumferential cell intercalation to thin and elongate the tubules [80].

### 3.5 Tip cell anchorage and looping morphogenesis

The analysis of tube elongation in branching structures tends to focus on growth and extension but many tubular systems adopt a mature looped structure, as in the salivary gland and nematode gonad and tubule looping is a characteristic feature of renal tubules from worms to man. However until recently rather little was known about how looped morphology is achieved.

Using the renal tubules of *Drosophila*, we have shown that tip cell activity determines the looped architecture of the anterior pair of tubules. Tip cells make transient contacts with dorsal muscles (the alary muscles) that anchor the heart. They contact these muscles at abdominal segment boundaries, moving progressively forwards as cell intercalation lengthens the tubule. Tip cells make a final target, always the alary muscle at segment A3/4 boundary, and this contact persists into the adult fly. As the tubules elongate they encounter guidance cues provided by TGF-β ([81]; see Fig. 3D). Tip cell anchorage antagonises this forward-directed tubule movement thereby ensuring both a looped morphology and the highly reproducible positioning of the tubules in the body cavity in relation to other tissues. If tip cells are ablated the looped structure is lost and tubules extend both too far anterior and ventrally in the body [12].

Distinctive tip cell filopodial activity, exploratory behaviour and specific cell adhesion, through the cell adhesion molecule Neuromusculin and integrins underlie the serial attachment of tip cells to successive alary muscles. Increased adhesion, either by the over-allocation of tip cells or by enhancing their expression of adhesion molecules results in persistence of the first, more posterior tip cell-muscle contact so that the tubules remain in the posterior of the body cavity. Basement membrane clearance over the tip cell surface by the repression of matrix deposition, expression of MMPs and transcytosis of matrix and their receptors by tip cells underlies target recognition and the dynamic interactions of these cells. Defects in these features obliterate tip cell anchorage, producing misshapen and misplaced tubules, which have impaired physiological function [12].

The role of tip cells in regulating tube architecture by anchorage to a distal muscle appears to be a conserved feature during the organogenesis of renal systems in diverse species. In the renal systems of other insects, including the cranefly *Ptychoptera* and another Drosophilid, *D. funebris*, the distal-most tip of each anterior tubule is attached to a fine striated branch of the dorsal alary muscles [82,83]. More recent studies of ‘tip cells’ in the excretory system of the Planarian flatworms have revealed intriguing parallels between the role of tip cells in these disparate systems [29]. The Planarian excretory system is composed of multiple protonephridial epithelial tubes that are capped at their tip by specialised flame cells. Electron microscopy reveals that flame cells display prominent filopodia and attach to nearby muscle fibres [29,84,85]. Given that flame cell loss appears to correlate with the collapse of tubule architecture [29], it has been suggested that tip cell anchorage to the surrounding muscular layer might play an important role during branching morphogenesis in this system.

A striking example of looped tubules is found in the mammalian kidney, where the distal and proximal convoluted tubules together with the loop of Henle connects the tubule tip (at the glomerulus) to the collecting duct (close to the site of urine outflow). Looping of both the nephron and its vascular supply creates a counter-current system that maximises the efficiency of ion and fluid homeostasis. Both the site of connection to the ureter and the tubule tip, the renal corpuscle, are established early in organ development so that tubule extension occurs between these fixed points [86]. Whether tubule tips play a prominent role in maintaining the looped structure as kidney tubules extend is not known; for example, if the developing tubule were to loop in response to guidance cues as it extends, the establishment of these distal and proximal fixed points would be key to the maintenance of a looped architecture.

### 3.6 Fusion at the tips: creating tubular networks

In the development of both the insect tracheal system and vertebrate vasculature, sets of discontinuous tubular elements must be connected to generate a fully functional tubular network.

In the *Drosophila* tracheal system, fusion cells are specified at the distal ends of the tracheal branches and act to interconnect independent or distant tracheal tubes (Fig. 3D). The processes underlying fusion are described by Luschnig in this issue and include fusion cell contact through their dynamic filopodial activity, E-cadherin-mediated adhesion, followed by cell contraction and apical membrane deposition to form a continuous lumen [13,87,88]. Very similar events occur during anastomosis (blood vessel fusion) in the vertebrate vasculature [89,90]. Indeed recent real-time *in vivo* imaging studies in zebrafish are beginning to elucidate the cellular mechanisms that govern vascular anastomosis [91].

Whilst the renal tubules of *Drosophila melanogaster* remain as four separate tubes throughout the life of the animal, the two posterior renal tubes of another Drosophilid, *D. funebris*, fuse at their distal ends to generate a single looped posterior structure around the hindgut [82]. This raises the possibility that the tip cells at the distal ends of each tube fuse to interconnect the two separate renal tubules in a similar way to the fusion cells of the tracheal system.

### 3.7 Tip cells as ‘master regulators’ in tubulogenesis

Clearly tip cells have a wide variety of functions but an emerging theme is that in many organs they perform multiple roles. For example the DTCs in the *C. elegans* gonad have a role in the maintenance of germline mitoses but also guide the outgrowth of the gonadal tubes by responding to a complex network of attractive and repulsive extracellular guidance cues released from dorsal and ventral body muscles along the route of migration ([32,40,41,43]; reviewed in [92]) and by secreting the MMP GON-1 to clear a migratory route [93,45].

The activity of tip cells in the development of *Drosophila* renal tubules demonstrates the versatility of tip cells; as in the worm gonad, Malpighian tubule tip cells regulate mitosis in neighbouring cells, they act to polarise these cells once cell division is complete (thereby regulating tubule extension by oriented cell movements), they act to explore the environment, responding to guidance cues to contact a series of target cells (thereby stabilising tubule architecture during morphogenesis) and they finally make a permanent specific contact, which maintains the shape and positioning of the mature tubule in the body cavity [12,35,94,80].

The distinctive characteristics of tip cells that enable them to regulate such a range of cell activities during tubulogenesis derives from their ability to act as sensors as well as regulators. They respond to signals, for example in responding to high levels of signalling as they promote branching in tracheal/vascular systems or in the recognition of specific cues for tissue guidance or for attachment to target tissues. However equally important is their ability to send signals (mitogenic signals, polarising signals or those for target recognition), to transmit mechanical stress (in tubule extension) and to modify the environment through the secretion of specific factors or by directed uptake and transcytosis of specific cargoes (such as MMPs or polarised endocytosis and transport to clear the overlying basement membrane).

This range of tip cell activity clearly depends on changes in their patterns of gene expression and deployment of the resulting gene products. While both mitogenesis and tissue polarity result from the release of EGF from the renal tip cell lineage, tubule architecture is regulated by distinctive, stage-specific filopodial activity, clearing of overlying basement membrane components, cell recognition of targets and the expression of tightly controlled levels of adhesion molecules. While it is likely that transcription of many tip cell specific genes is downstream of the AS-C, which acts to specify tip cells [8], the precise relationship between cell specification and later tip cell patterns of gene expression (such as those encoding MMPs and integrins; [12]) is not yet known.

The versatility of renal tubule tip cells depends not only on these changes in tip cell activity but also on alterations in the responding cells. It is striking that the tip cell signal, EGF, controls mitosis during embryonic stages 11–12 and that the same signal regulates the planar polarised activity of Myosin II in the same, now post-mitotic, responding cells from stages 13–16 [35,80]. Activation of the EGF pathway that leads to mitosis is known to act through transcriptional regulation [36,37]; the response to later signalling that alters cytoskeletal activity and cell movement is not fully understood but could involve post translational modifications of proteins that are already expressed in responding cells [80]. Finally positioning of the anterior renal tubules depends on the balance of antagonistic forces between those derived from tip cell-alary muscle adhesion which antagonise forward-directed movement generated by tubule cell intercalation, and response to pathfinding cues.

## 4 Tip cell roles in tubule physiology

The tip cells of many organs, including the insect tracheal system and the mammalian vasculature, remain active post-embryonically and modulate tissue structure in response to changes in organismal physiology or environmental conditions. This is particularly important during periods of hypoxia, where tip cells promote the growth of new tubular branches to adapt to local oxygen availability [27,28,95] and for review see [96]. The response of tracheal cells is discussed by Luschnig (in this issue).

There is currently no evidence that the insect renal system is extensively remodelled post-embryonically. However, renal tip cells remain attached to their muscle and neuronal target tissues throughout larval and adult life [8,12], suggesting possible physiological roles. Persistence of anterior tip cell attachment to alary muscles anchors the distal tubule ends and maintains the normal distribution of tubules in the body cavity, which is important to maximise the efficiency of tubule function during osmotic/ionic homeostasis and excretion [12]. Similarly, the renal tubule tips in crickets such as *Acheta domestica* are anchored to the body wall to ensure the hundreds of tubules are well dispersed throughout the haemocoel [97]. Alary muscles are contractile and so agitate the tubules in the haemolymph, increasing fluid sampling and possibly increasing the paracellular flow of solutes (such as novel toxins) into the tubule lumen for excretion.

## 5 Concluding remarks

Tip cells play pivotal roles in multiple ways during normal tubule development as well as in their physiological responsiveness. We have reviewed the defects that arise when their normal activities are compromised; for example without tip cells fly renal tubules contain half the normal number of cells and the short, stumpy tubules fail to elongate or take up their normal positions in the body cavity. As a result, the clearance of toxins from the haemolymph as well as ionic and water balance are severely compromised [98,99]. Failure in normal tubulogenesis is associated with many congenital disease conditions, such as polycystic kidney disease and the development of fistulas. Further, the growth of solid tumours depends on their vascularisation, through angiogenic sprouting that parallels the normal response to hypoxic signals. Clearly a detailed understanding of tubulogenesis and the factors that regulate the cellular processes that underlie it, including the activity of tip cells, is important for our understanding of healthy development, physiological responsiveness and for the treatment of disease.
